# Looking back on 51 years of the Carol Nachman Prize in Rheumatology—significance for the field of spondyloarthritis research

**DOI:** 10.1007/s00393-024-01496-w

**Published:** 2024-06-12

**Authors:** Jürgen Braun, Joachim Sieper, Elisabeth Märker-Hermann

**Affiliations:** 1 Rheumatologisches Versorgungszentrum Steglitz, Schloßstr. 110, 12163 Berlin, Germany; 2 Rheumatologie am Campus Benjamin Franklin, Medizinische Klinik für Gastroenterologie, Infektiologie und Rheumatologie, Charité Universitätsmedizin Berlin, Berlin, Germany; 3 DKD Helios Klinik Wiesbaden GmbH, Wiesbaden, Germany

**Keywords:** Spondyloarthritis, Psoriatic arthritis, Reactive arthritis, HLA-B27, TNF alpha, IL-17, Spondyloarthritis, Psoriasisarthritis, Reaktive Arthritis, HLA-B27, TNF-alpha, IL-17

## Abstract

The city and casino of Wiesbaden, capital of the German state Hessen, have endowed the Carol Nachman Prize to promote research work in the field of rheumatology since 1972. The prize, endowed with 37,500 €, is the second highest medical award in Germany and serves to promote clinical, therapeutic, and experimental research work in the field of rheumatology. In June 2022, the 50-year anniversary was celebrated. In the symposium preceding the award ceremony, an overview was given on the significance of spondyloarthritis for the work of the awardees in the past 30 years. This overview has now been put together to inform the interested community of the work performed, including the opinion of the awardees regarding what they consider to be their most important contribution.

## Abstract (de)

Die Stadt Wiesbaden, Landeshauptstadt von Hessen, und die Spielbank Wiesbaden stiften seit 1972 den Carol-Nachman-Preis, um die Forschung auf dem Gebiet der Rheumatologie zu fördern. Der mit 37.500 € dotierte Preis ist damit die zweithöchste medizinische Auszeichnung in Deutschland, er dient der Förderung klinischer, therapeutischer und experimenteller Forschung im Bereich der Rheumatologie. Im Juni 2022 wurde das 50. Jubiläum gefeiert. In dem Symposium, das der Preisverleihung voranging, wurde ein Überblick über die Bedeutung der Spondyloarthritiden für die Arbeit der Preisträger in den vergangenen 30 Jahren gegeben. Die vorliegende Übersicht ist zusammengestellt worden, um alle Interessierten über die geleisteten Arbeiten der Preisträger zu informieren und umfasst auch deren Meinungen in Bezug auf das, was sie jeweils selbst als ihre wichtigste Leistung auf dem Gebiet der Spondyloarthritiden ansehen.

## Introduction

The casino (*Spielbank*) of Wiesbaden, capital of the German state of Hessen, has endowed the Carol Nachman Prize to promote research work in the field of rheumatology since 1972. Since 1987, a Carol Nachman Medal has also been awarded. The prize, endowed with 37,500 euro, is the second highest medical award in Germany and serves to promote clinical, therapeutic, and experimental research work in the international field of rheumatology. The Carol Nachman Prize and Medal bear the name of their donor, the long-time casino concessionaire and honorary citizen of Wiesbaden, Carol Nachman. About 50 years ago, he and the Wiesbaden rheumatologist Prof. Klaus Miehlke launched the prize together with the Mayor of Wiesbaden at that time, Alfred Herbel. Since 1972, the prize has been awarded to more than 80 internationally recognized scientists. The prize is awarded based on evaluation of their work by an independent international scientific committee.

Even after the death of the prize donor, the casino of Wiesbaden has continued to provide the financial support. The aim of the annual endowment of the Wiesbaden State Capital Prize for Rheumatology is to honor the work of medical doctors and basic scientists in combating these widespread diseases, which is so valuable for everyone. Over the years, the casino has provided more than 1.6 million euro for this purpose.

The city of Wiesbaden sees itself as a “rheumatism city” because internationally renowned medical professionals treat thousands of patients with rheumatic diseases every year in internal rheumatology and orthopedic hospitals, outpatient clinics, and the specialist rheumatology practices of the city. In addition, scientific training congresses bring many rheumatologists here, and not only during the annual internal medicine congress in April. For the casino of Wiesbaden, this is reason enough to support the awarding of the “Prize of the State Capital Wiesbaden for Rheumatology” with great commitment on an ongoing basis.

On the afternoon before the award ceremony, the Carol Nachman Symposium “50 years of the Carol Nachman Prize—50 years of milestones in rheumatology” was organized by the chairperson of the board of trustees, Elisabeth Märker-Hermann, who has already served in this position since 2010 following the late Prof. Joachim R. Kalden. Internationally renowned speakers commemorated 50 years of milestones in rheumatology (epidemiology and public health research, rheumatoid arthritis, spondyloarthritis, and systemic lupus erythematosus) at the ceremonial hall of Wiesbaden City Hall. The talk of Prof. Jürgen Braun serves as the basis for this overview of the Carol Nachman Prize related to the field of spondyloarthritis during the past roughly 30 years.

## Methods

For the 50th anniversary of the Carol Nachman Prize of the City of Wiesbaden, which was celebrated on June 23, 2022, a number of previous prizewinners were invited to hold lectures. Among others, J. Braun was asked to create an overview of the award winners of the previous 30 years in the field of spondyloarthritis (SpA). On the basis of the list of prizewinners provided, a selection was made as to which prizewinners had made a name for themselves either primarily or also in the field of SpA and had published important articles. Then the winners were emailed and asked to name their 5–8 most important publications in this field. In the reference list, the names of Carol Nachman Prize winners are highlighted in bold.

## Results

The awardees are listed in Table 1.

**Table 1:** List of awardees of the Carol Nachman Price

Year	Names	Country	City	Main area of research
1992	Robert Hammer Joel Taurog	USA	Dallas, Texas	HLA-B27 transgenic rat model, effects of a germ-free environment
2000	Juergen Braun Joachim Sieper	Germany	Berlin	IBP, MRI, NSAID, and anti-TNF therapy T cell immunology, immunohistology, cohort studies
2003	Robin Poole	Canada	Toronto	T cell immunology, autoimmunity, antigen cross-reactivity
2004	Auli Toivanen Paavo Toivanen	Finland	Turku	Role of bacteria in ReA, efficacy of antibiotics in ReA
2010	Pierre Miossec	France	Lyon	Cytokine networks, Th17 pathway
2011	Désirée van der Heijde	Netherlands	Leiden	Epidemiology, classification criteria, disease activity score, therapy studies
2012	Paul Emery	England/UK	Leeds	MRI, NSAID, and anti-TNF therapy
2015	Georg Schett	Austria	Erlangen	Osteoimmunology, role of bone in SpA
2016	Maxime Dougados	France	Paris	Epidemiology, classification criteria, cohort studies, T2T in SpA
2018	Dafna Gladman	Canada	Toronto	Psoriatic arthritis, cohort studies
2019	Ian McInnes	Scotland/UK	Glasgow	T cell immunology, Th17
2021	Dirk Elewaut	Belgium	Gent	Role of mechanical stress, the gut, and the microbiome in SpA
2022	Maria D’Agostino	Italy	Rome	Ultrasound for diagnosis and monitoring of SpA
2023	Martin Rudwaleit	Germany	Bielefeld	ASAS criteria, concept of axSpA, IBP
2023	Denis McGonagle	Ireland	Leeds	Enthesitis, immunologic concepts, PsA

*MRI* magnetic resonance imaging; *TNF* tumor necrosis factor; *ASAS* Assessment of SpondyloArthritis International Society

A short introduction to their work and their main publications is presented below. Usually, 5–8 publications were given.

## Carol Nachman Price awarded in 1992

### Prof. Robert Hammer and Prof. Joel Taurog, University of Texas Southwestern Medical Center, Department of Biochemistry, University of Texas Southwestern Medical Center in Dallas, Division of Rheumatic Diseases

The main part of their work related to spondyloarthritis (SpA) is based on a really fascinating animal model. Their first paper described this animal model showing that transgenic rats expressing HLA-B27 and human beta 2 microglobulin develop a disease with similarities to human SpA [1]. In the same model, it was shown that expression of HLA-B27 correlates with the SpA-like symptoms developing in the animals [2]. Furthermore, the authors showed that the SpA-like disease could be transferred by bone marrow engraftment [3]. Another important finding and certainly among the most interesting findings of that model was that the SpA-like disease did not develop if the rats were held in a germ-free environment [4]. Furthermore, the authors demonstrated that normal luminal bacteria, especially *Bacteroides *species, may function as mediators of chronic colitis, gastritis, and arthritis in HLA-B27/human beta 2 microglobulin transgenic rats [5].

Although the question remains as to what this model has finally taught us about the human disease, this was great scientific work that included major genetic and immunologic research questions.

## Carol Nachman Price awarded in 2000

### Prof. Joachim Sieper and Prof. Jürgen Braun, Charité University Medicine, UKBF Berlin, Germany

The work of these authors also included many aspects of SpA but they concentrated on human material such as synovial fluid and sacroiliac biopsies and modern imaging technology. Their first important study showed that magnetic resonance imaging (MRI) is useful to detect active sacroiliitis in patients with no definite radiographic changes [6]. In a landmark study, the authors demonstrated for the first time that tumor necrosis factor alpha (TNFα) is heavily expressed in inflamed sacroiliac joints of patients with ankylosing spondylitis (AS, [7]), now named axial spondyloarthritis (axSpA). In a follow-up study, the degree of enhancement detected by MRI was shown to correlate with cellularity in early and active sacroiliitis in patients with axSpA, [8]. The next project was a pilot study that showed for the first time that anti-TNF therapy (with infliximab) works very well in patients with AS [9]. In the following first randomized controlled trial on biologics in axSpA it was clearly demonstrated that inhibitors of TNF (TNFi) are efficacious in AS [10]. This study provided the basis for approval of infliximab for AS by the European Medicines Agency (EMA), because of an unmet need in this disease. These studies provided the main basis for being awarded with Carol Nachman Prize and later, in 2003, the European League Against Rheumatism (EULAR) award.

In the over 20 years after receiving the Carol Nachman Prize, these two authors have continued to contribute considerably in different fields of spondyloarthritis such as diagnosis, new treatments, imaging, and outcome parameters.

### Prof. Joachim Sieper, Charité Universitätsmedizin Berlin, UKBF

In an important early study, the authors showed that the T cell cytokine pattern detected in the synovial membrane of patients with rheumatoid arthritis (RA) and reactive arthritis (ReA) differs regarding the expression of cytokines such as interferon gamma (IFNγ) and interleukin (IL)‑4 [11] according to the T helper cell (Th)1/Th2 paradigm [12].

More than 10 years later, the Assessment of SpondyloArthritis International Society (ASAS) group developed candidate criteria for the classification of axSpA including patients without radiographic changes in the sacroiliac joints [13]. Thereafter, the first classification criteria for axSpA, which are still used, were validated [14].

These classification criteria, in conjunction with the 1984 modified New York criteria [15], allowed for differentiation between radiographic (r-axSpA) and non-radiographic (nr-axSpA) axSpA. The former is largely equivalent to AS [16]. However, nr-axSpA developed as a new indication for biologic and targeted synthetic disease-modifying anti-rheumatic drugs (b- and tsDMARDs). The first agent to be successfully studied in this indication of nr-axSpA was the TNFi adalimumab [17]. Neglecting the difference between r‑axSpA and nr-axSpA, which is only relevant for classification and not for clinical diagnoses, another important study in early, active axSpA compared infliximab plus the non-steroidal antiinflammatory drug (NSAID) naproxen versus naproxen alone in a double-blind, placebo-controlled trial, INFAST [18]. Expectedly, infliximab worked better but naproxen also led to remission in about 30% of patients (as compared to about 60% with the TNFi).

Shortly thereafter, it became clear that there are other bDMARDs working in AS: of those blocking interleukin 17 (IL-17), the first one approved for AS was secukinumab [19], and later ixekizumab. Both agents are also efficacious in nr-axSpA, as published in early 2020 [20]. As of today, five TNFi are approved for axSpA (only infliximab is not approved for nr-axSpA), and three (infliximab, etanercept, adalimumab) have already several biosimilars approved. In addition, there are, in the meantime, three IL-17i (secukinumab, ixekizumab), and recently also bimekizumab [21]. Furthermore, there are also two janus kinase (JAK) kinase inhibitors, tofacitinib and upadacitinib approved in the field of SpA [22]. Great achievements by large national and international studies were made in which this awardee has often played a leading role.

### Prof. Jürgen Braun, Rheumazentrum Ruhrgebiet 2001–2021, Ruhr Universität Bochum, 2024 Rheumatologisches Versorgungszentrum Steglitz

After having developed a new MRI-based scoring system to quantify spinal inflammation in patients with AS [23], the authors were able to demonstrate for the first time in a multicenter, randomized, double-blind, placebo-controlled trial that treatment with infliximab led to a major reduction of axial inflammation in the vertebral column [24].

The relationship between inflammation and new bone formation in AS was analyzed in detail to show that inflammatory MRI changes precede new bone formation in AS [25].

Many years later, a large population-based study taking advantage of MRI performed as part of the Study of Health in Pomerania (SHIP) was conducted. In this project, the frequency of MRI changes suggestive of axSpA in the axial skeleton in a large cohort of individuals aged < 45 years was assessed and found to be much more frequent than previously thought [26]. Furthermore, the study results supported the hypothesis of mechanical strain contributing to the presence of bone marrow edema (BME) in the general population aged < 45 years and the role of HLA-B27+ as a severity rather than a susceptibility factor for BME in the sacroiliac joints [27].

After long discussions on how to define severity, the ASAS decided to develop a health index for patients with AS, the ASAS HI [28]. This global initiative based on the International Classification of Functioning, Disability, and Health (ICF) developed by the World Health Organization (WHO) allows for quantification of global functioning of patients with AS.

Some years later, the ASAS developed quality standards to improve the quality of health and care services of patients with axSpA [29].

The treat-to-target strategy (T2T) has been increasingly recognized as the best way to treat inflammatory rheumatic diseases. It is associated with a strong tendency to focus on disease activity markers and the reduction of inflammation. In an important review [30] about the significance of physical function and activity in axSpA, it is stressed that rheumatologists should also consider these outcome parameters as important in their management strategy to treat patients with axSpA—which is very consistent with the quality standards for axSpA [29].

## Carol Nachman Price awarded in 2003

### Prof. A. Robin Poole, McGill University, Montréal, Canada

This author has mainly concentrated on autoimmune responses to the connective tissue structure of cartilage. The matrix of cartilage contains glycosaminoglycans, proteoglycans, and collagen fibers. Immunity to proteoglycans can indeed be induced by injection of human cartilage proteoglycan in BALB/c mice, since these animals develop progressive polyarthritis and AS-like features [31]. More specifically, such rheumatic symptoms could also be induced in these mice by the proteoglycan aggrecan G1 domain. In this animal model, a T cell line specific to an epitope on the G1 domain of aggrecan induced arthritic symptoms by adoptive transfer and homing to the intraarticular epitope [32]. Furthermore, the results of proliferation assays with peripheral blood lymphocytes from patients and healthy controls suggested that the cartilage link protein is a potential autoantigen in the development of both RA and AS [33].

When this link protein was repeatedly injected intraperitoneally into BALB/c mice, a persistent, erosive, inflammatory polyarthritis developed. Importantly, a single T cell epitope was recognized by specific T lymphocytes [34]. Immunity to cartilage molecules such as link protein, aggrecan, or the G1 domain of aggrecan has also been observed in patients with AS. In an interesting review, it was also proposed that involvement of other tissues involved in axSpA such as the eye and the aorta may be due to cross-reactive immunity [35].

Also here, an important research question related to immunity to human cartilage was tackled by studies mainly using an animal model but also with human peripheral blood. However, as of today, no more evidence has been generated to support that hypothesis.

## Carol Nachman Price awarded in 2004

### Profs. Auli und Paavo Toivanen, University of Turku

These authors have concentrated on the role of bacteria in the pathogenesis of ReA, which constitutes a small part of the spectrum of SpA. ReA causes joint pain and swelling, most often asymmetric, in knees, ankles, and feet triggered by an infection, usually caused by bacteria such as *Chlamydia, Salmonella*, and *Yersinia* in another part of the body, most often in the gastrointestinal or the urogenital tract. A major research question has always been whether the presence of microbes or a pathologic reaction of the immune system is more important for the pathogenesis. Using polymerase chain reaction (PCR), chromosomal DNA of *Yersinia* was not found in the synovial specimen of patients with *Yersinia*-triggered ReA or controls [36]. However, with immunocytochemical techniques, *Yersinia* antigens were observed in synovial specimens from patients with *Yersini*a‑triggered ReA [37]. Later work demonstrated for the first time that bacterial antigens may persist for a long time in patients who develop ReA after an infection with *Yersinia enterocolitica* O:3 [37]. The *Yersinia* adhesin, YadA, seems to be involved in interactions with extracellular matrix molecules after invasion of the intestinal tissue [38].

In a first study to assess bacteria-specific immune responses in HLA-B27+ compared to HLA-B27− individuals, antibodies to *Yersinia, Salmonella*, and *Klebsiella* were more often found in the former, suggesting that differences in such immune responses are related to HLA-B27 [39].

In an important first study using an animal model, it was shown that antibiotic therapy of *Yersinia*-triggered ReA only works if the treatment is started very early in the course of the disease and if given in sufficient dosage [40]. However, antibiotic treatment had no effect on fully developed arthritis and also not on antibody formation [41].

As of today, the detection of chromosomal bacterial material in the synovial fluid has a place in clinical practice especially for *Chlamydia*, but this often does not take place because the incidence of ReA has rather declined in recent decades. Similarly, antibiotic therapy will especially be performed if there is evidence of *Chlamydia *in the urogenital tract.

## Carol Nachman Price awarded in 2010

### Prof. Pierre Miossec, University of Lyon, France

The inhibitory effect of soluble TNF receptors on IL‑6 production and collagen degradation in synovium and bone was shown to be increased upon adding soluble IL-17 receptor and soluble IL‑1 receptor II. This supports the concept of combination therapy to further increase the response to therapy [42]. Later, it became clear that Th17 cells produce IL-17, and that IL-17 is inhibited by IFN‑γ, while IL-23 enhances IL-17 production. Furthermore, there was increasing evidence that IL-17 is able to induce IL‑1 and TNF production, while IL‑1 and IL‑6 can increase IL-17 secretion but IL‑4 inhibits IL-17 [43]. The Th17 subset is a new T cell subset [44] described in addition to the Th1 and Th2 T cell subsets, which seems to be controlled through IL-23 [45]. These Th17 cells became more and more likely to play a key role in chronic inflammatory diseases. Treatment trials several years later made it clear that IL-17 does not play an important role in RA but rather in psoriasis and in axSpA. Based on recent clinical trial data, IL-23 and IL-17 are at least partly uncoupled in axSpA. Reasons as to why, when, and how this plays a role in the pathogenesis of SpA were discussed with special reference to the microenvironment of the subchondral bone marrow. Especially the different interactions between lymphocytes and stromal cells play an important role in immune responses [46]. Stromal cells have indeed been shown to contribute to inflammation—from induction to chronicity or resolution—through direct cell interactions and through the secretion of pro-inflammatory and anti-inflammatory mediators. Today, anti-IL17 therapy has an established place in the treatment of axSpA and psoriatic arthritis (PsA).

## Carol Nachman Price awarded in 2011

### Prof. Désirée van der Heijde, University of Leiden, the Netherlands

This author is the most important and leading figure of ASAS who has initiated many projects to standardize measurements and instruments to assess important features of AS and axSpA. In a very early project [47], instruments for the core set for DC-ART, SMARD, physical therapy, and clinical record-keeping in AS were selected by the ASAS Working Group to be able to compare results across studies. This core set has recently been updated [48].

The studies leading to establishment of the classification criteria for axSpA which are now used worldwide in many trials have already been cited [13, 14]. Another landmark study led to establishment of the disease activity criteria for axSpA which are now also used worldwide in clinical studies, the ASDAS [49]. Another very important study linked disease activity to radiographic progression in axSpA [50]. The ASAS-EULAR management recommendations for axSpA, which have led rheumatologists all over the world on how to treat their patients [51], have recently been updated [52]. Great achievements by large national and international studies were made in which this awardee has often played a leading role.

## Carol Nachman Price awarded in 2012

### Prof. Paul Emery, University of Leeds, England, UK

This author has a great record in many fields of rheumatology, with significant contributions also in the field of SpA. In an early review, we were reminded of the significance of enthesitis in axSpA [53]. Unlike monoclonal antibodies, the TNF receptor fusion protein etanercept works on arthritis but not on colitis in Crohn’s-related SpA [54]. Later, it became clear that the frequency of flares of Crohn’s disease was also higher in axSpA patients treated with etanercept [55].

In a long-term study, it was shown that the degree of inflammation in the SIJ in combination with HLA-B27 were predictive of structural changes in these joints in the further course of patients with axSpA [56].

In a landmark study with very early young HLA-B27+ patients with axSpA treated with the TNFi infliximab, partial remission was reached in more than half of the cases [57]. In another landmark study, it was shown that a T2T approach leads to better outcomes with a few more side effects in PsA [58]. The T2T approach is nowadays more and more established, also in the field of SpA. Enthesitis is an important outcome in clinical studies. Great achievements by large national and international studies were made in which this awardee has often played a leading role.

## Carol Nachman Price awarded in 2015

### Prof. Dr. Georg Schett, Universität Erlangen

This author has made important basic science and clinical contributions not only in the field of SpA. Among the first was the Dickkopf (DKK)-related protein 1 that is encoded in humans by the *DKK1* gene. DKK‑1 inhibits the Wnt signal transduction pathway. Wnt signaling is a multifaceted pathway that regulates several important cellular pathways which are β‑catenin-dependent or not. TNFα was identified as a key inducer of DKK‑1 in a mouse inflammatory arthritis model and also in human RA. These results suggested that the Wnt pathway is a key regulator of joint remodeling [65].

Psoriasis patients without arthritis show substantial signs of enthesophyte formation, representing new bone formation at mechanically exposed sites of joints. This finding supports the concept of a deep Koebner phenomenon at entheseal sites in patients with psoriasis [66].

The pathophysiology of enthesitis is of special interest in the field of SpA. In an important review, the role of biomechanics, prostaglandin E2-mediated vasodilation, and the activation of innate immune cells in the initial phase of enthesitis was addressed [67], and also the possible role of entheseal IL-23-responsive proinflammatory cells that produce IL-17, IL-22, and TNFα.

The data of another study strongly suggested that a very early disease interception in patients with incipient psoriatic arthritis leads not only to a decline in skin symptoms but also to reduction of pain and subclinical inflammation [68]. Finally, in a highly cited review, the ability to block specific cytokine pathways was interpreted as an important tool to reveal pathophysiological differences among autoimmune diseases, hereby providing a framework for reclassification of rheumatic diseases [69].

## Carol Nachman Price awarded in 2016

### Prof. Maxime Dougados, René Descartes University of Paris, Hospital Cochin, France

This author has a great record in many fields of rheumatology, with significant contributions in the field of SpA. In the first randomized placebo-controlled clinical trial to study the efficacy and safety of a potential disease-modifying anti-rheumatic drug (DMARD) in AS, Sulfasalazin, there was no convincing evidence of its efficacy [59]. After the first attempt to enlarge the spectrum of SpA [60], ASAS improvement criteria, initially for AS, were developed [60], which are still widely used.

Following the German early SpA inception cohort GESPIC [61], the DESIR (DEvenir des Spondylarthropathies Indifférenciées Récentes) cohort is one of the most important cohort studies in the field of axSpA. An early study showed that HLA-B27 is associated with earlier onset of IBP, less delay in diagnosis, more axial inflammation, and more radiographic changes in the SIJ [62].

The ASAS-COMOSPA study focused on comorbidities and included almost 4000 patients worldwide. The most frequent comorbidities found were osteoporosis and gastroduodenal ulcer, and the most frequent risk factors hypertension, smoking, and hypercholesterolemia, indicating a significant cardiovascular risk of these patients [63]. In another landmark T2T study, it was shown that this approach led to better outcomes than traditional strategies in patients with axSpA [64]. Great achievements by large national and international studies were made in which this awardee has often played a leading role.

## Carol Nachman Price awarded in 2018

### Prof. Dafna Gladman, University of Toronto, Canada

This author has worked intensively all her life on all aspects of PsA. In an early study it was shown that PsA can be a rather severe disease [70]. The role of environmental factors in the development of PsA was highlighted in another study [71], while the disadvantages of late presentation of patients with PsA were stressed by showing that this is associated with more joint damage [72]. The contribution of certain HLA‑B and HLA‑C alleles to the susceptibility to PsA among patients with psoriasis was analyzed in another study [73], while the biomarkers metalloproteinase (MMP)-3 and cartilage oligomeric matrix protein (COMP) were found to be predictive of drug responses to anti-TNF therapy in patients with PsA [74].

The prospective follow-up of patients with psoriasis was instrumental to show that the chemokine CXCL10 is a biomarker for the development of PsA [75]. Furthermore, a preclinical phase was described that is characterized by nonspecific musculoskeletal symptoms, including joint pain, fatigue, and stiffness, which was found to precede the diagnosis of PsA in patients with psoriasis [76].

## Carol Nachman Price awarded in 2019

### Prof. Iain McInnes, University of Glasgow, Scotland, UK

As already pointed out, the authors of this review argued that “immune-mediated inflammatory diseases” should be less defined by their organ involvement but rather by a molecular-based classification [69]. Indeed, a similar response or non-response to targeted anti-cytokine therapies might be more connecting than an organ-based definition [77].

The role of dendritic cells (DC) was highlighted based on the finding of a reduced number of these cells in peripheral blood (PB) of RA and PsA patients, an increase in synovial fluid compared to PB, and an incomplete maturation of these cells in the inflamed synovial compartment [78].

In an important head-to-head (H2H) trial in PsA, a similar proportion of ACR20 responders was found among patients treated with the JAK inhibitor upadacitinib dosed at 15 mg/day and the TNFi adalimumab at 40 mg every 2 weeks, while the response rates for the 30 mg/day dose of upadacitinib was even higher than for adalimumab. However, the higher dose has not been approved for this indication. Both drugs were superior to placebo [79].

In another H2H trial, the IL-17i secukinumab was not superior to adalimumab in patients with active PsA. The higher retention rate of secukinumab could be due to the superior effect of the IL-17i on skin manifestations [80]. The anti-IL23 antibody guselkumab, which binds selectively to the p19 subunit of this cytokine, was much more efficacious than placebo in a large randomized controlled trial in active PsA [81]. Great achievements by large national and international studies were made in which this awardee has often played a leading role.

## Carol Nachman Price awarded in 2020/2021 (due to COVID-19 pandemic)

### Prof. Dirk Elewaut, University of Ghent, Belgium

This author has made important basic science contributions to the field of SpA. In an animal model, the role of mechanical stress was shown to be mandatory for inflammation and new bone formation at enthesial sites and probably also at other sites of the axial skeleton, providing some explanations regarding the sites of disease manifestations in SpA [82]. More evidence that mechanical strain controls the site-specific localization of inflammation and tissue damage in arthritis was provided in a following study [83].

Dysregulated IL-23/IL-17 responses seem to be present in SpA. The retinoic acid receptor-related orphan receptor gamma isoform t (RORγt) is an important Th17 cell transcriptional regulator. The potential of ROR-γt antagonism to modulate aberrant type-17 responses was highlighted in a study with SpA patients [84].

In work focusing on the microbiome, it was shown that the intestinal microbial composition of patients with SpA who have microscopic gut inflammation is different compared to those without colitis. Moreover, the microbial genus *Dialister *was abundantly present in the gut of these patients, which correlated with disease activity [85]. In recent decades, the role of gut inflammation has been studied a lot—not only in Gent. In this important study, an association between gut inflammation and the degree of subchondral bone marrow edema in the sacroiliac joints of SpA patients gave new support to the long-postulated gut–joint link in this disease [86].

## Carol Nachman Price awarded in 2022

### Prof. Maria Antonietta D’Agostino, Universita Cattolicà del Sacro Cuore, Rome, Italy

This author has mainly focused on imaging in SpA. In an early cross-sectional study, power Doppler ultrasonography was introduced for investigation of SpA-associated peripheral enthesitis [87]. Some evidence was provided that this technique can be helpful to diagnose SpA early in a follow-up study [88]. The frequency of synovitis was determined in an ultrasound study in the healthy population. The findings were found to be relevant for calculation of the specificity of a positive ultrasound finding in the diagnostic approach of SpA [89]. By analyzing data from the DESIR cohort on patients with early axSpA, the authors identified two clinical phenotypes—one with predominantly axial manifestations and one with predominantly peripheral manifestations [90].

In the context of an OMERACT project, a final reliable ultrasound score and a consensus-based definition of enthesitis in SpA were generated, with possible implications for clinical practice [91].

In a therapeutic study, the treatment response of PsA patients to secukinumab was studied by ultrasonography which showed a rapid reduction of synovitis, in good correlation to improvement of clinical symptoms [92].

In a follow-up study of an earlier investigation, it was shown that the presence of peripheral manifestations at diagnosis of axSpA results in a poorer clinical outcome compared to an axial manifestation alone [93].

## Carol Nachman Price awarded in 2023

### Univ.-Prof. Dr. med. Martin Rudwaleit and Prof. Dr. Denis McGonagle; Univ.-Prof. Dr. med. Martin Rudwaleit, University of Bielefeld, Germany

This author has not only worked on the definition of IBP [94, 95], but also very much on the diagnosis and classification of axSpA [13, 14, 96–98]. Regarding anti-TNF therapy, he has made clinically relevant contributions for the prediction of a major response to this treatment [99, 100]. Following the early study on the efficacy of NSAIDs to reduce radiographic progression in AS showing that continuous treatment with celecoxib is superior to on demand therapy [101], he and his coworkers studied the performance of diclofenac in this regard but failed to show an effect [102]. However, in contrast, a dose-dependent effect of smoking on radiographic progression in AS was clearly demonstrated [103]. Currently, M. Rudwaleit is leading the ASAS-part of the CLASSIC study, an international multicenter trial including a second evaluation of the ASAS classification [13, 14] criteria, aiming for an increase in their specificity.

### Prof. Dr. Denis McGonagle, University of Leeds, UK

This author, last but clearly not least, started early by reminding us that enthesitis is an important clinical feature of SpA [53, 104]. The late J. Ball had expressed his view on the relevance of enthesitis in his Heberden Oration lecture [105] shortly before the association of AS with HLA-B27 had been discovered 50 years ago (as recently reviewed [106]). Nevertheless, this author has taken a deep dive into the synovio-entheseal complex and he has nicely explained the functional interdependence of an enthesis with adjacent synovium and how this has an influence on the phenotypic expression of joint disease—not only in PsA [107].

An important step in the understanding of immune-mediated diseases was his paper on the differentiation of autoimmunity from autoinflammation [108]. The recognition and genetic understanding of autoinflammatory diseases has helped to define mechanisms of self-directed inflammation which act independently of adaptive immunity. Local factors at sites predisposed to disease lead to activation of innate immune cells such as macrophages and neutrophils. For example, disturbed homeostasis of canonical cytokine cascades (as in periodic fever syndromes), aberrant bacterial sensing (as in Crohn’s disease), and tissue microdamage (as in PsA) predispose to site-specific inflammation triggered by innate immune dysregulation at sites of mechanical stress, driving SpA pathology [108].

Fitting into this concept, the author later proposed together with Turkish colleagues the term “MHC-I-opathy” [109] to explain how and why Behçet’s disease and several clinically distinct forms of SpA, all associated with MHC class I alleles such as *HLA-B-51, HLA-C-0602*, and *HLA-B-27* and epistatic *ERAP1* interactions, have a shared immunopathogenetic basis. This also includes a barrier dysfunction in environmentally exposed organs such as the skin, and aberrant innate immune reactions at sites of mechanical stress [109].

Finally, taking advantage of human spinous processes, entheseal soft tissue, and peri-entheseal bone harvested during elective orthopedic procedures, the author showed that the spinal entheseal Vδ1 and Vδ2 subsets are tissue-resident cells with inducible IL-17A production [110]. He also provided evidence that the Vδ1 subset does so independently of IL-23R expression. This is important because a sophisticated animal experiment had shown several years ago that IL-23 is essential in enthesitis by acting on a previously unidentified IL-23 receptor [111]. However, there was some disappointment later on because therapies directed against IL-23 did not work in AS [112], while anti-IL-17 antibodies did [19]. The situation became even more complicated when it became clear that anti-IL-23 but not anti-IL-17 agents work in IBD, while both were shown to be efficacious in psoriasis and PsA [69].

Very recently, McGonagle proposed an explanation for differences between axSpA and PsA with axial involvement, arguing that in contrast to “HLA-B27-associated disease,” the enthesitis seen in PsA primarily manifests in ligamentous soft tissue as “ligamentitis” [113]. This may also explain differential responses to IL-23 inhibition, since the enthesis bone and soft tissues have radically different immune cell and stromal compositions.

## Discussion

This article was prepared to give an overview of the work of the Carol Nachman Prize winners and their impact on spondyloarthritis research. The list of awardees and their most important publications provides an overall view of their personal achievements during the past decades. About 40% of the awardees of the Carol Nachman Prize in the last 31 years were working at least in part in the field of spondyloarthritis.

The following themes were covered:

- Axial spondyloarthritis
- Psoriatic arthritis
- Reactive arthritis
- Enthesitis
- Inflammatory bowel disease
- Mechanical stress as trigger
- Autoimmunity
- Microbiome
- IL-17/IL-23
- Osteoimmunology
- Comorbidity
- Magnetic resonance imaging
- Ultrasound
- Radiographic progression

The above list is an interesting mixture of different fields in medicine and rheumatology covering clinical science, epidemiology, imaging, therapy, basic research, cytokines, animal models, the microbiome, autoimmunity, and mechanical factors. For the majority of the award winners cited here, the field of spondyloarthritis was the focus or at least a major part of larger scientific projects in basic immunology and rheumatology. However, several authors have also contributed significant work to the pathogenesis of rheumatoid arthritis and osteoarthritis or to the epidemiology and management of other rheumatic diseases. Looking at the reference list, most authors have worked together in many projects. This may well be one reason for the great success of research in rheumatology and the field of SpA.

Of the 18 awardees presented here, 4 were women (22%) and 14 men (78%). This imbalance should be considered as a stimulation for more female researchers in the field of rheumatology. In fact, studies on sex- and gender-related differences regarding diseases and treatment outcomes have substantially increased in the past years.

The main reason for the absence of Carol Nachman Prize winners in the field of spondyloarthritis before 1992 mainly indicates that the focus of international and national rheumatology was rather on rheumatoid arthritis and connective tissue diseases. The discovery of the HLA-B27 association by D. Brewerton [114], the proposed concept of spondyloarthritis by J. Moll & V. Wright [115], the first description of inflammatory back pain by A. Calin [116], the first hint that NSAIDs may inhibit radiographic progression in AS [117], the detailed radiographic concept of radiographic changes in the sacroiliac joint by W. Dihlmann [118], the distinguished and still valid calculation of the diagnostic performance of HLA-B27 by M. Khan [119], and the publication of the modified New York criteria for AS by S. van der Linden [120] are good examples of valuable scientific research in the field of AS.

The award of the Carol Nachman Prize and the annual celebration in the city of Wiesbaden has always been a festive event to appreciate prior work or even lifetime achievement of the awardees and their groups. In addition, it has been a challenge to generate new knowledge and promote research in rheumatology. Awards such as the Carol Nachman Prize have contributed and continue to contribute a lot to the stimulation of research in rheumatology.

## Declarations

For this article no studies with human participants or animals were performed by any of the authors. All studies mentioned were in accordance with the ethical standards indicated in each case.
